# Multidisciplinary management promoting wound healing in a patient with Proteus syndrome: a case report

**DOI:** 10.3389/fped.2026.1800678

**Published:** 2026-06-15

**Authors:** Yue Tan, Xu Zhang, Hai-Bin Wang, Li Wang, Wen-Shuang Qiu

**Affiliations:** 1 Clinical Pharmacy Department, Affiliated Hospital of Jining Medical University, Jining, China; 2 Department of Orthopedics, Affiliated Hospital of Jining Medical University, Jining, China; 3 Pharmacy Department, Affiliated Hospital of Jining Medical University, Jining, China

**Keywords:** congenital abnormalities, infection, pediatric disease, pharmacist, proteus syndrome

## Abstract

**Introduction:**

Proteus syndrome is caused by AKT1 gene mutations. It manifests during childhood and results in asymmetric overgrowth of skin, bone, central nervous system, and adipose tissues and has a poor survival rate. Currently, there are no targeted drug treatments for this condition. Due to its rarity, the nonspecific symptoms and complications of Proteus syndrome often go unrecognized and, thus, untreated by clinicians.

**Case description:**

This case report documents the diagnosis and treatment of a 14-year-old patient with Proteus syndrome who developed severe soft tissue infection secondary to lower limb deformity. A clinical pharmacist implemented individualized anti-infective interventions based on etiology, liver function, and body weight, significantly optimizing the treatment course and promoting wound healing.

**Discussion:**

Through this report, we aim to enhance awareness of the diverse clinical presentations of Proteus syndrome and emphasize the need for a coordinated healthcare approach to effectively manage its associated complications.

## 1 Introduction

Proteus syndrome (PS) is a rare disorder, affecting approximately 1 in 10 million individuals (1). First described by the German pediatrician Rudolf Wiedemann in 1983, PS typically affects young children, presenting soon after the age of one, and involves progressive, asymmetric overgrowth of the skin and bones (2, 3). Cerebriform connective tissue nevi or plantar cerebriform collagenomas occur in 67% of patients with PS and are the most distinctive skin manifestations of this syndrome (4). Plantar lesions can hinder walking and often require surgical intervention. Furthermore, PS is associated with severe orthopedic issues, deformities, and increased mortality (5). Conventional treatment focuses on symptom management and does not alter disease progression (6). In 2011, researchers identified that the genetic mutation responsible for PS is an activating mutation in serine/threonine kinase 1 (AKT1) in mosaic cells.

The PI3K/AKT/mTOR pathway is crucial for the regulation of growth, metabolism, and survival of normal cells. Somatic mutations in genes involved in this pathway, such as activating mutations of PIK3CA, AKT1, and mTOR and inactivating mutations of PTEN and TSC1/2, lead to various overgrowth syndromes with overlapping phenotypes. These mutations are associated with conditions such as PS and PIK3CA-related overgrowth spectrum (PROS) (7). Abnormal activation of this pathway can compromise the skin barrier and alter the local immune microenvironment and blood supply, therefore patients are more susceptible to infections that rapidly spread, making treatment challenging and slowing healing. However, there are few reports on targeted therapeutic agents. The safety and tolerability of the selective pan-AKT inhibitor miransertib in patients with PROS or PS were evaluated in a multicenter open-label phase I/II MOSAIC study (NCT03094832). Miransertib was generally well-tolerated, with most drug-related adverse events being low grade. No drug-related deaths or discontinuations occurred. However, its efficacy has not been assessed using standard imaging endpoints. Notably, only four patients with PS were included, and the long-term safety data were limited, but the researchers observed a median treatment duration of 20.5 months (8). Additionally, there have been no direct comparisons of targeted drugs. The somatic activating mutation in AKT1 c.49GA (p.Glu17Lys) has a mutant allele ratio ranging from 1% to 50%. This mutation leads to abnormal cell proliferation by activating the PI3K-AKT pathway (9–12), which supports the hypothesis of somatic mosaic mutations (13). Although single case reports have identified mutations in the PI3K-AKT pathway, comprehensive studies are lacking.

Currently, reports of coinfection management in PS remain limited. In this report, we discuss the management of a lower-limb deformity and skin infection in a 14-year-old male patient with PS. This case report aims to enhance clinical management and improve outcomes for patients with PS.

## 2 Case description

A 14-year-old boy presented to our clinic with fever, a skin ulceration on the right lower limb, and rapidly spreading erythema and edema extending to the thigh. The right lower limb exhibited a pronounced hypertrophic deformity, accompanied by cerebriform plaques on both soles. The patient had been diagnosed with PS (Supplementary Figure S1). Since childhood, he had undergone numerous orthopedic procedures. His skin deteriorated and became infected because of the abnormal development and protrusion of the ankle bone, extending to the right thigh. This infection was characterized by erythema, edema, discomfort, and elevated skin temperature. On the first day after admission, the inflammatory indicators (white blood cell count: 4.7 × 10⁹/L, neutrophil percentage: 88.1%, CRP: 113.1 mg/L, procalcitonin: 0.526 ng/mL) and liver function (aspartate aminotransferase: 112.3 U/L, *γ*-glutamyl transpeptidase: 29.0 U/L) were measured. The resident doctor prescribed cefotiam 2 g q12h. On the second day, liver damage was confirmed. The pharmacist suggested using liver-protecting drugs, therefore silybin meglumine tablets, glutathione, and bifendate dropping pills were added to the treatment regime. On the fourth day, the patient's inflammatory indicator levels were measured (white blood cell count: 6.9 × 10⁹/L, neutrophil percentage: 88.8%, CRP: 104.0 mg/L, procalcitonin: 0.624 ng/mL). The results of bacterial culture + drug sensitivity indicated Streptococcus pyogenes, which was sensitive to penicillins, resistant to clindamycin and ceftriaxone, and sensitive to vancomycin. On the seventh day, the patient had recurrent high fever. After analyzing the poor anti-infection effect, the clinical pharmacist suggested adjusting the drug doses. The clinical pharmacist optimized the anti-infection regimen according to drug sensitivity, severity of infection, body weight, and liver function, and changed the drug to oxacillin 2 g, q8h administered intravenously, which is suitable for adolescents with a body weight > 40 kg. On the eighth day, the inflammatory indicator levels were re-examined, and the results were white blood cell count: 8.92 × 10⁹/L, neutrophil percentage: 79.7%, CRP: 49.9 mg/L, and procalcitonin: 0.127 ng/mL.Consequently, the patient showed symptoms of improvement and was discharged 7 days after antibiotic treatment modification (Supplementary Figure S2).

Examination of wound exudates using microbiological cultures identified the occasional presence of gram-positive cocci and Streptococcus pyogenes. On the basis of the results and the patient's weight of > 40 kg, the pharmacist advised a dosage of 2 g of oxacillin every 8 h. Although *Staphylococcus aureus* was not detected, the pharmacist's assessment indicated its ubiquitous existence on the human skin. The pharmacist emphasized that with impaired skin barriers, *S. aureus* can easily invade wounds (14). The patient had no adverse drug reactions, such as rash, nausea, or abdominal pain during treatment, and showed good tolerance.

## 3 Discussion

Pharmacological therapy for PS remains rarely reported in clinical trials. In a previous case report, a 7-year-old patient with early-stage PS was treated with sirolimus for six years, resulting in a 19.1% reduction in the area of cerebriform connective-tissue nevus, stable skeletal deformities, mild drug-related adverse effects, and an acceptable safety profile (15). PS is a progressive disorder marked by abnormal tissue proliferation caused by dysregulated PI3K/AKT/mTOR signaling, leading to extensive tissue abnormalities. This case presents a treatment strategy for severe orthopedic issues compounded by infection.

This case report is the first to document an incidence of PS concurrent with a soft-tissue infection in the lower limb, with infection treatment managed by a pharmacist. To address current clinical demands, clinical pharmacists should be integrated into medical and nursing teams to actively contribute to condition management as well as drug treatment services. This is particularly crucial in pediatric pharmacy services, as rational drug use in children is essential. Children diagnosed with bacterial infections or those at risk of infections require close monitoring. Customized monitoring plans should be established to assist clinics in assessing the infection location, severity, potential pathogens, and appropriate drug indications. In one previous case, a child with a severe infection could not undergo surgery because of rapid disease progression. If infections are not addressed promptly, children may experience serious consequences, e.g., becoming ineligible for important surgeries. Pharmacists play vital roles in selecting and dosing medications, planning treatment, and ensuring precise and consistent care. They can adjust doses based on a child's liver and kidney function and collaborate with physicians to enhance drug treatment while minimizing the risk of adverse reactions.

The patient in this case had symptoms of PS acquired in early childhood, with pronounced skeletal abnormalities in the lower limbs and cerebriform structures in both feet. Owing to their significant effect on his quality of life, he had undergone multiple surgical operations. However, a secondary infection at the ankle had rapidly spread to the right leg. Without adequate infection management, more severe complications such as sepsis, deep muscle infection, and extensive persistent infection may develop, thereby precluding surgical intervention. Thus, a multidisciplinary approach to treatment planning is essential to avert the development of such complications, and meticulous drug management is paramount in conservative therapy. The patient and his family members were satisfied with the treatment outcome and he resumed normal daily activities. Cases such as this are infrequently documented, with no recorded treatment results for similar conditions. Therefore, this case report offers novel data for the management of patients with similar presentations.

Compared with soft tissue infections in healthy children, infections in patients with PS are more likely to spread rapidly, involve deep tissues, respond poorly to conventional treatments, heal more slowly, and have a higher risk of recurrence. This case suggests that for patients with PS complicated by infections, simple empirical anti-infective treatment yields poor results. Individualized medication must be implemented in combination with etiological results, organ function, and disease characteristics. Compared with the traditional physician-led empirical treatment, the precise intervention of clinical pharmacists can significantly improve the effectiveness and safety of treatment. This case further confirms that multidisciplinary collaboration is an effective model for managing the complex complications of PS. However, due to being limited by the single-case observation and lack of AKT1 gene verification, the conclusions of this study need to be further verified in larger-scale, multi-center studies. In the future, multi-center case series studies should be conducted to provide higher-level evidence for the standardized management of infectious complications in PS.

## Data Availability

The original contributions presented in the study are included in the article/Supplementary Material, further inquiries can be directed to the corresponding author.
