# Supplementary material for: Multidisciplinary management promoting wound healing in a patient with Proteus syndrome: a case report
Source: Front Pediatr. 2026 Jun 15;14:1800678. doi: 10.3389/fped.2026.1800678 (PMC13311120; doi:10.3389/fped.2026.1800678)
Supplement: Supplementary file 1 [file Supplementaryfile1.docx]

Supplementary Material

# Supplementary Figures


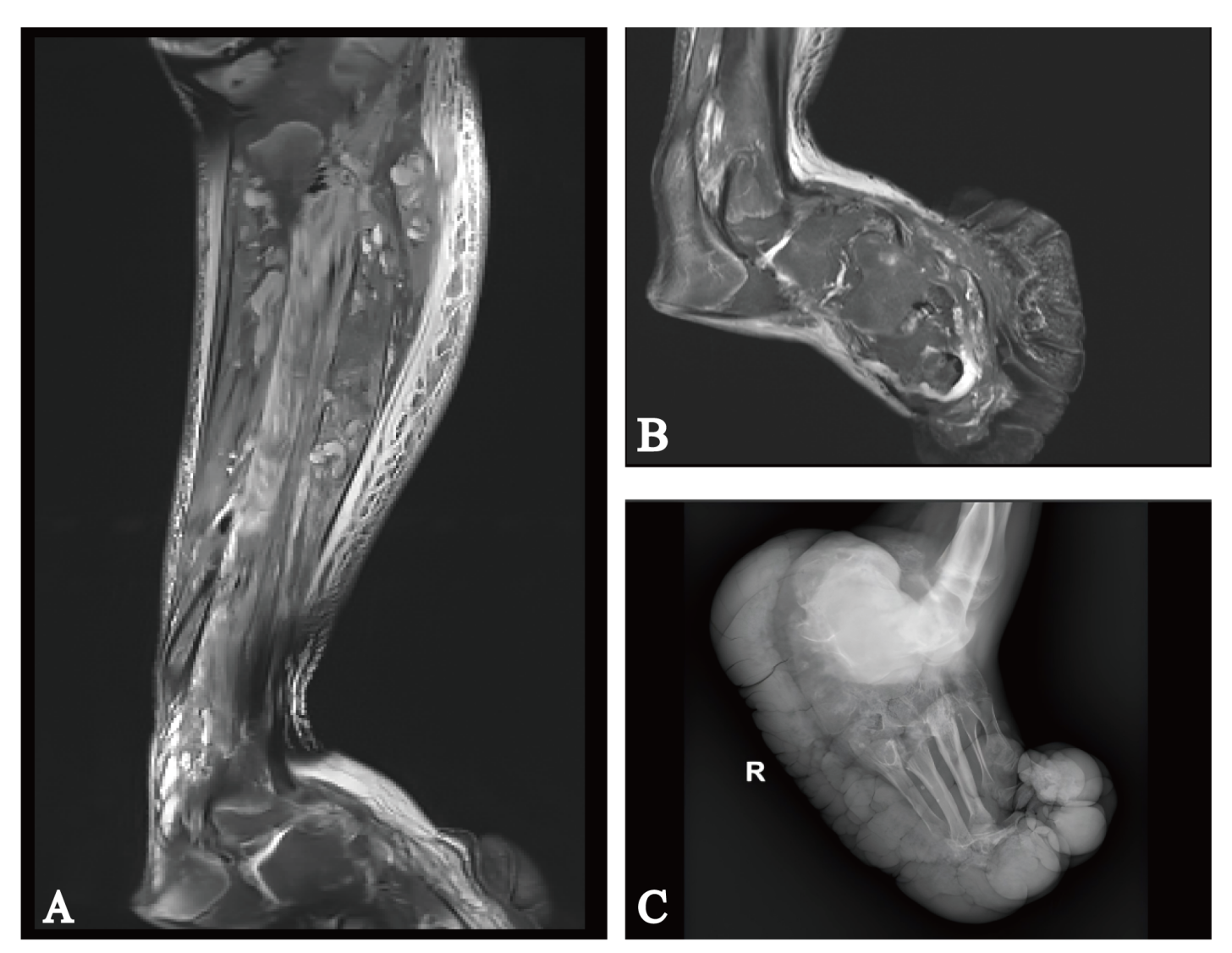


FIGURE 1. MRI: The right femur and right tibia and fibula exhibit irregular morphology, thickening, and elongation. The right knee, ankle, and foot joints show deformities accompanied by multiple bony protrusions and hypertrophy. In conjunction with the clinical history, Proteus syndrome is considered **(A-B)**. X-ray: The second metatarsal bone of the right foot is slender and elongated, while the first metatarsal is relatively short; the distal ends of the phalanges are thick and hammer-shaped **(C)**.


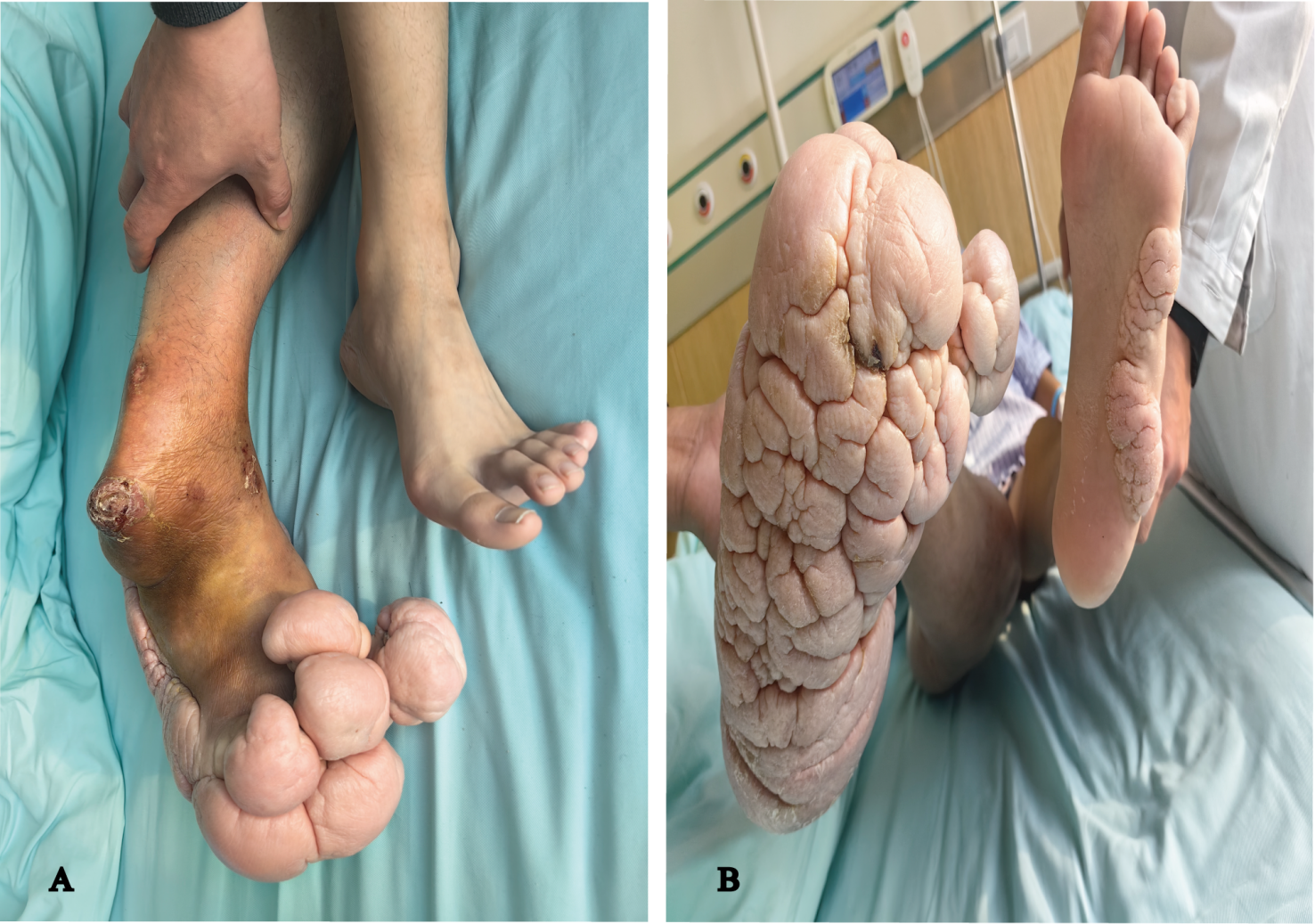


FIGURE 2. Recovery of cerebral convoluted connective tissue nevus and skin soft tissue after treatme **(A-B)**
